# Heterologous expression of the isopimaric acid pathway in *Nicotiana benthamiana* and the effect of N-terminal modifications of the involved cytochrome P450 enzyme

**DOI:** 10.1186/s13036-015-0022-z

**Published:** 2015-12-22

**Authors:** Thiyagarajan Gnanasekaran, Konstantinos Vavitsas, Johan Andersen-Ranberg, Agnieszka Zygadlo Nielsen, Carl Erik Olsen, Björn Hamberger, Poul Erik Jensen

**Affiliations:** Department of Plant and Environmental Sciences, Copenhagen Plant Science Centre, UNIK Center for Synthetic Biology, Villum Research Center “Plant Plasticity”, University of Copenhagen, Thorvaldsensvej 40, DK-1871 Frederiksberg C, Copenhagen, Denmark; Present address: Plant and Microbial Biology, University of California, 371 Koshland Hall, Berkeley, CA 94720 USA

**Keywords:** Chloroplasts, Cytochrome P450, Diterpenoids, Isopimaric acid, Protein engineering

## Abstract

**Background:**

Plant terpenoids are known for their diversity, stereochemical complexity, and their commercial interest as pharmaceuticals, food additives, and cosmetics. Developing biotechnology approaches for the production of these compounds in heterologous hosts can increase their market availability, reduce their cost, and provide sustainable production platforms. In this context, we aimed at producing the antimicrobial diterpenoid isopimaric acid from Sitka spruce. Isopimaric acid is synthesized using geranylgeranyl diphosphate as a precursor molecule that is cyclized by a diterpene synthase in the chloroplast and subsequently oxidized by a cytochrome P450, CYP720B4.

**Results:**

We transiently expressed the isopimaric acid pathway in *Nicotiana benthamiana* leaves and enhanced its productivity by the expression of two rate-limiting steps in the pathway (providing the general precursor of diterpenes). This co-expression resulted in 3-fold increase in the accumulation of both isopimaradiene and isopimaric acid detected using GC-MS and LC-MS methodology. We also showed that modifying or deleting the transmembrane helix of CYP720B4 does not alter the enzyme activity and led to successful accumulation of isopimaric acid in the infiltrated leaves. Furthermore, we demonstrated that a modified membrane anchor is a prerequisite for a functional CYP720B4 enzyme when the chloroplast targeting peptide is added. We report the accumulation of 45–55 μg/g plant dry weight of isopimaric acid four days after the infiltration with the modified enzymes.

**Conclusions:**

It is possible to localize a diterpenoid pathway from spruce fully within the chloroplast of *N. benthamiana* and a few modifications of the N-terminal sequences of the CYP720B4 can facilitate the expression of plant P450s in the plastids. The coupling of terpene biosynthesis closer to photosynthesis paves the way for light-driven biosynthesis of valuable terpenoids.

## Background

Plant specialized metabolites comprise a large pool of compounds that play a major role in functions such as interaction with the environment, defense, and stress tolerance [1]. Diterpenoids— C-20 terpenes that number more than 12,000 identified metabolites [2]—represent a considerable part of this chemical wealth. In addition, they have a wide range of applications as pharmaceuticals, cosmetics, and food additives [3–6]. In plants, the initial steps of the diterpenoid biosynthesis occur in the plastids, where diterpene synthases (diTPS) catalyze the cyclization of geranylgeranyl pyrophosphate (GGPP) [5, 7]. Diterpenoids display a remarkable diversity in terms of structure and physiological role; this chemical variety initiates from the amount of carbon skeleton arrangements the diTPS can produce [2, 7], and which is further augmented by cytochrome P450s [8].

Cytochrome P450s are a versatile enzyme superfamily that performs stereo-specific oxygenation via an electron-consuming, heme-dependent catalytic mechanism [9]. In plants, the majority of the P450s are found in the endoplasmic reticulum (ER) anchored via their N-terminal membrane domain, although some P450s are found soluble in the mitochondria and chloroplasts [10, 11]. Expressing a plant P450 in bacteria or yeast is not straightforward, due to issues such as codon usage and membrane localization of the protein [12]. Another limiting factor is the availability of reducing power in heterologous hosts [3, 13]. Recently, it was shown that plant P450s can use electrons directly from photoreduced ferredoxin, both *in vitro* and *in vivo* [14–19].

**Fig. 1:**
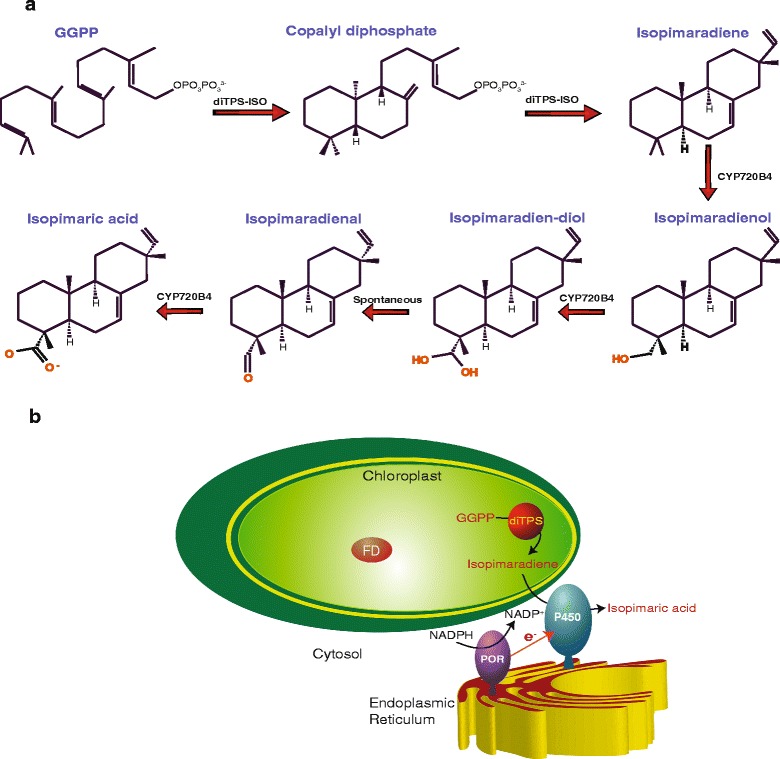
Isopimaric acid biosynthesis and localization of the participating enzymes. **a** Biosynthetic pathway of isopimaric acid from GGPP. The five enzymatic steps are catalyzed by diTPS-ISO and CYP720B4. **b** Schematic representation of the localization of the enzymes involved in the isopimaric acid pathway. DiTPS-ISO is located in the chloroplast, where it cyclizes GGPP to isopimaradiene, which is subsequently oxygenated by CYP720B4 in the ER. The electrons required by the CYP720B4 (CYP) are provided by a P450 oxido-reductase (POR) by oxidizing NADPH. The generic redox cofactor of the chloroplast ferredoxin (FD) is also indicated

Isopimaric acid is a diterpene resin acid produced by conifers. It is a defense compound that prevents spore germination of the pathogenic fungus *Ophiostoma ips* and affects the feeding habits of gypsy moth (*Lymantria dispar*) larvae and various sawfly species [20–22]. Isopimaric acid is also reported to have antibacterial activity against multidrug resistant *Staphylococcus aureus* strains that can cause bacteremia, endocarditis and hemolytic pneumonia in humans [23]. In Sitka spruce, the isopimaric acid pathway involves only two enzymes, a bi-functional diterpene synthase, isopimara-7, 15-diene synthase (diTPS-ISO) and a multifunctional cytochrome P450, CYP720B4. The diterpene synthase converts GGPP to the tricyclic diterpene isopimara-7, 15-diene (isopimaradiene). In three subsequent oxygenation reactions, the ER-localized CYP720B4 catalyzes the conversion of isopimaradiene into isopimaric acid (Fig. 1) [24–26].

In this work, we studied the expression of the isopimaric acid pathway in *Nicotiana benthamiana* leaves using transient infiltration with *Agrobacterium tumefaciens*. Plant chloroplasts are attractive bioengineering targets: they already serve as biosynthetic sites for various metabolites, they provide a prokaryotic-like environment in terms of protein expression and regulation, and they offer the opportunity to directly exploit the reducing power of photosynthesis [3, 27, 28]. We modified the N-terminal part of the CYP720B4 in order to target it to the chloroplast and we assessed our engineering approaches via immunoblotting and metabolite analysis.

## Results and discussion

### *In-vivo* production of isopimaradiene and isopimaric acid in *N. benthamiana*

**Fig. 2:**
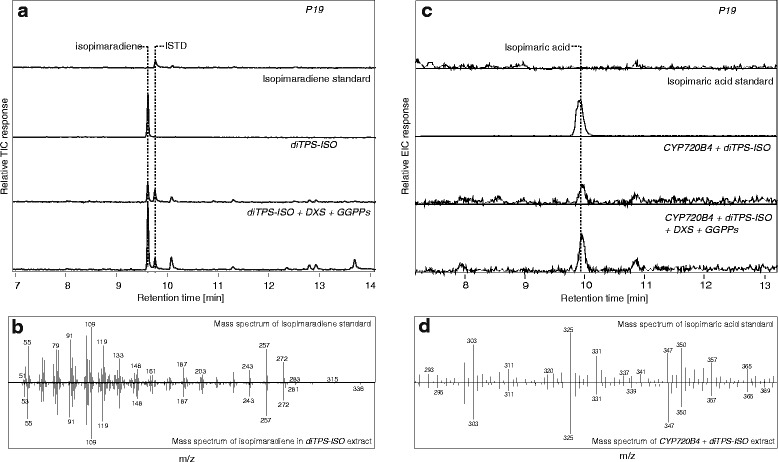
Metabolite analysis of infiltrated plant leaves. **a** Total Ion Chromatograms (TIC) for the detection of isopimaradiene using GC-MS analysis. Samples were spiked with the internal standard Eicosene (ISTD). **b** Mass spectra of isopimaradiene detected in the standard and *diTPS-ISO* infiltrated leaf extracts. **c** Extracted ion chromatograms (m/z −303[M + H]^+^, 325[M + Na]^+^, 347 [M + 2Na]^+^) for the detection of isopimaric acid using LC-MS analysis. Isopimaric acid is detected at retention time 10 min. **d** Mass spectra of isopimaric acid detected in the standard and *CYP720B4* + *diTPS-ISO* infiltrated leaf extracts

To express the entire isopimaric acid pathway, we infiltrated *N. benthamiana* plants with *A. tumefaciens,* harboring plasmids encoding both *diTPS-ISO* and *CYP720B4. P19* silencing repressor gene-infiltrated plants [29] were used as controls. The expressed diTPS-ISO is active as confirmed by the formation of isopimaradiene in the infiltrated leaves (Fig. 2a, b) (for expression of the diTPS-ISO protein in the infiltrated leaves refer to the following section). Upon co-expression with CYP720B4, isopimaradiene was depleted and isopimaric acid accumulated (Fig. 2c, d), indicating efficient conversion. The *P19* blank infiltration samples displayed a background free of isopimaradiene and isopimaric acid.

To improve the production of isopimaric acid, we utilized the 1-deoxyxylulose-5-phosphate synthase (DXS) and GGPP synthase (GGPPs) from *Plectranthus barbatus* (Indian coleus). Those two enzymes belong to the GGPP biosynthetic pathway and are considered rate-limiting for diterpenoid production [30, 31]. The co-expression of DXS and GGPPs together with the isopimaric acid enzymes led to a 3-fold increase in the accumulation of both isopimaradiene and isopimaric acid (Fig. 2a, c). We overexpressed the enzymes of *P. barbatus,* instead of the native ones, in order to avoid any feedback regulation and post- translational modifications that adjust the DXS and GGPPs activity [32–34]. In a previous study from our lab (Randberg et al., unpublished), we showed that expressing DXS and GGPPs from *P. barbatus* in *N. benthamiana* were efficient in boosting diterpenoid levels compared to co-expression of the native DXS and GGPPs genes, which had no effect on boosting diterpenoid level.

**Fig. 3:**
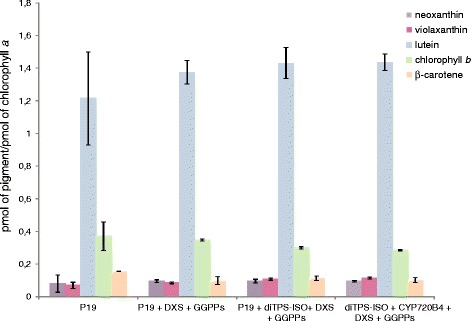
Pigment analysis of infiltrated leaves. P19-infiltrated plants were used as controls. The increased expression of DXS and GGPPs during the transient expression experiments did not result in significant variations in pigment composition. Pigments quantities are given relative to chlorophyll *a*. Biological triplicates were used for the quantification

Rerouting GGPP into novel diterpenes might have an effect on native GGPP-derived metabolites or pigments. We measured the relative quantity of the photosynthetic pigments chlorophyll *a*, chlorophyll *b*, violaxanthin, neoxanthin, lutein, and β-carotene by performing high performance liquid chromatography (HPLC). Comparing infiltrated leaf extracts and controls showed no detectable difference (Fig. 3).

### Expression of N-terminal modified CYP720B4 in *N. benthamiana*

**Fig. 4:**
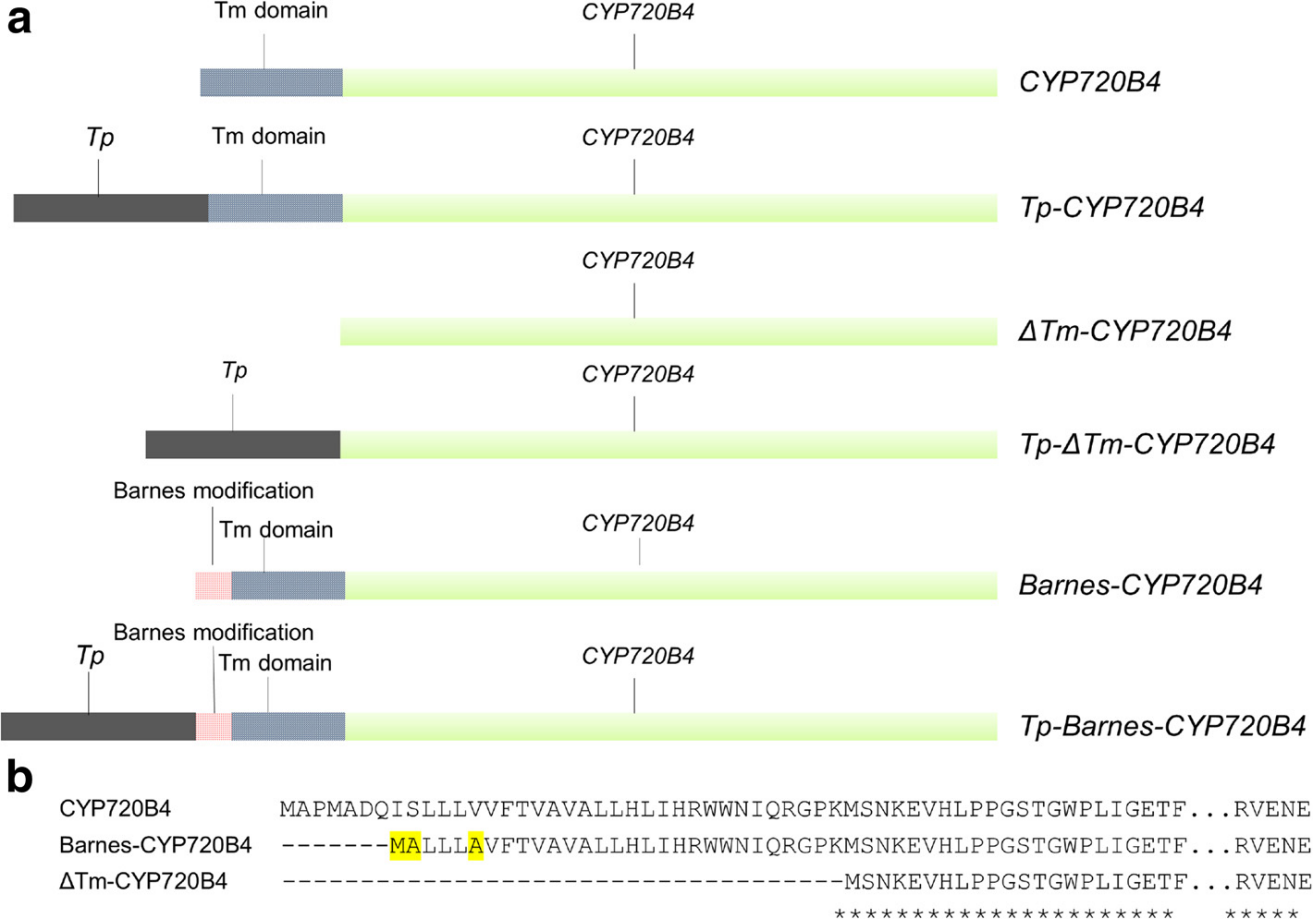
*CYP720B4* modifications used in this work. **a** Schematic representation of *CYP720B4* sequences, where the catalytic part of the protein sequence is depicted in green, while the N-terminal parts are noted in more detail: Tm domain = Transmembrane domain of the CYP720B4;  *Tp*  - Transit peptide (the N-terminal 52 amino acid of the Arabidopsis ferredoxin protein FedA); and Barnes modification  - omission the first 21 bases of the coding sequence and introduction of three point mutations in the N-terminal part. **b** Protein sequence alignment of the N-terminal parts of CYP720B4, Barnes-CYP720B4 and ΔTm-CYP720B4. The point mutations of Barnes-CYP are highlighted in yellow

To express CYP720B4 in *N. benthamiana,* we used the native CYP720B4 coding sequence and two different CYP720B4 variants that were modified in the N-terminal membrane anchor domain (Fig. 4a). Initially, to investigate if the native membrane anchor of CYP720B4 is essential for its activity, we eliminated the N-terminal membrane domain of CYP720B4 (ΔTm-CYP720B4) by truncating the region that encodes the first 37 amino acids (Fig. 4b). We also implemented a strategy similar to the one used to express the human P450 17-alpha hydroxylase [35] and CYP720B4 [26] in *Escherichia coli*: we deleted 7 N-terminal amino acids from the membrane anchor (Δ3-9) and mutated a valine in position 13 to alanine (Barnes-CYP720B4) (Fig. 4b). Finally, to target the CYP720B4 enzyme to the chloroplasts, we made the following gene constructs variants Tp-CYP720B4, Tp-ΔTm-CYP720B4 and Tp-Barnes-CYP720B4 by fusing the coding region of the chloroplast transit peptide (Tp) of the *Arabidopsis thaliana* ferredoxin protein FedA [36] in the N-termini of CYP720B4, ΔTm-CYP720B4 and Barnes-CYP720B4 (Fig. 4a).

**Fig. 5:**
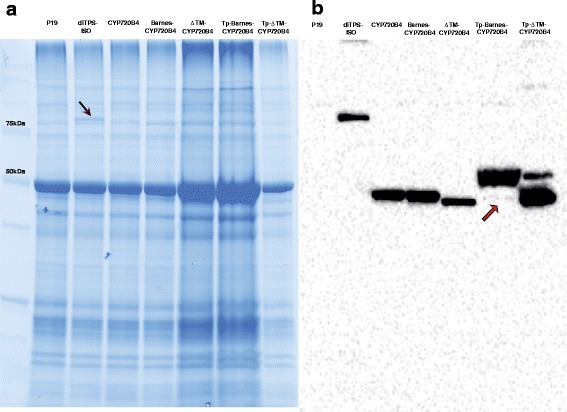
Analysis of protein expression in the transiently transformed leaves. Shown are SDS-PAGE (**a**) and immunodetection (**b**) of total protein extracts from crude leaf of infiltrated plants. Protein amounts corresponding to the total chlorophyll amount of 5 μg (P19, DiTPS-ISO, CYP720B4, and Barnes-CYP720B4), 10 μg (ΔTm-CYP720B4), 15 μg (Tp-Barnes-CYP720B4) and 3 μg (Tp-ΔTm-CYP720B4) were electrophoresed using SDS-PAGE. For immunodetection, Anti-FLAG antibody was used. The expected molecular masses of the FLAG-tag proteins DiTPS-ISO, CYP720B4, Barnes-CYP720B4 and ΔTm-CYP720B4 without the chloroplast transit peptide are 92, 55, 54 and 51 kDa respectively, and the masses of Tp-Barnes-CYP720B4 and Tp- ΔTm -CYP720B4 are 60 and 57 kDa respectively. The black arrow points to the visible protein band of DiTPS-ISO in SDS-PAGE. Red arrow points to faint immunodetection signal of Tp-Barnes-CYP720B4 that has presumably been processed upon transport into the chloroplasts

Immunoblots of the crude leaf extracts showed that CYP720B4 was expressed from all CYP720B4 construct variants (Fig. 5). Importantly, the enzymes that contained ferredoxin transit peptide (Tp-Barnes-CYP720B4 and Tp-ΔTm-CYP720B4) showed two protein bands, consistent in size with the precursor protein and the mature protein where the Tp was removed. Such a processing takes place during protein translocation into the plastid [37], corroborating the successful targeting of Tp-Barnes-CYP720B4 and Tp-ΔTm-CYP720B4 to the chloroplast.

### Effect of N-terminal modifications in CYP720B4 activity

**Fig. 6:**
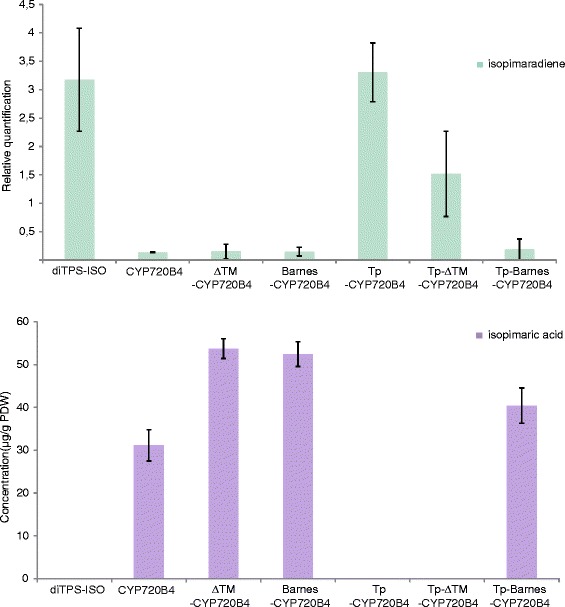
Quantification of **a** isopimaradiene and **b** isopimaric acid detected in infiltrated leaves. The bar labelled (diTPS-ISO) indicates the quantities of isopimaradiene/isopimaric acid produced upon co-expressing *diTPS-ISO*, *DXS* and *GGPPs* constructs. All other bars display the quantities of isopimaradiene/isopimaric acid produced upon co-expressing different *CYP* constructs with *DXS*, *GGPPs* and *diTPS-ISO*. Isopimaradiene is quantified relatively to the internal standard eicosene, and the isopimaric acid quantity was quantified relative to plant dry weight (PDW). Biological triplicates were used for the quantification

The diTPS-ISO and the CYP720B4 are naturally directed to the chloroplast and ER, respectively (Fig. 1b). CYP720B4 remains active even after complete removal of the transmembrane domain (Fig. 6); it is possible that either the remaining enzyme core can direct itself to the ER or that interaction with the P450 oxidoreductase allows proper localization. Truncated P450s do not generally lose the ability to associate with lipid membranes, however the ratio between ER-bound and soluble protein can change in favor of the latter [10, 38]. Barnes-CYP720B4 activity does not seem to deteriorate after the N-terminal modification (Fig. 6).

Most chloroplast proteins are encoded by the nucleus, hence they need to move into the organelle post-translationally [27]. Previously, CYP79A1 and CYP71E1*,* involved in the dhurrin biosynthetic pathway, have been functionally expressed in tobacco chloroplasts by fusing the N-terminus of the enzyme with the ferredoxin transit peptide [15]. We used the same approach in the present work, but the native CYP720B4 lost enzymatic activity when fused with the transit peptide (Fig. 6). In the dhurrin study [15], the P450 substrate was the hydrophilic compound tyrosine, while CYP720B4 acts on the hydrophobic isopimaradiene, which is a cyclic and non-oxygenated hydrocarbon (Fig. 1a). Taking this consideration one step further, we speculate that a proper membrane anchoring is crucial for CYP720B4 activity or substrate availability for the CYP720B4 active site. Membrane-bound and soluble P450s adopt different orientation of the heme within the protein [39], a fact that could result in major differences in the enzymatic properties.

The deletion of the transmembrane domain of CYP720B4 resulted in an active enzyme; however, the addition of the targeting peptide to Tp-ΔTm-CYP720B4 led to no product formation, despite the apparent high amount of processed enzyme detected in immunoblotting (Fig. 5b). In contrast, the Tp-Barnes-CYP720B4 was active. This suggests that the Barnes modification stabilizes CYP720B4 in the chloroplast. The large amount of unprocessed protein detected in the immunoblot could mean that a significant fraction of the enzyme remains in the cytosol or is ectopically inserted in the ER.

## Conclusions

Chloroplasts have already been engineered to over-produce carotenoids [40, 41] and have a large biotechnological potential that has been extensively reviewed [42, 43]. In this work, we showed that it is possible to localize a diterpenoid pathway from spruce fully within the chloroplast of *N. benthamiana*. We demonstrate that a few modifications of the N-terminal sequences of the CYP720B4, such as the modifications mentioned by Barnes and coworkers [35], can facilitate the expression of plant P450s not only in bacteria [26], but also in the plastids. Moreover, we assume that the membrane anchor is of importance for heterologous expression in algal chloroplasts or cyanobacteria, organisms that grow in liquid cultures and have potential as large-scale production systems [44].

P450s display various degrees of substrate promiscuity [45], a fact that can account for the terpenoid diversity, while it permits combinatorial approaches that differentially oxygenate the same cyclic skeleton [46]. Therefore, P450 enzymes are prominent candidates for synthetic biology exploitation, especially under the consideration that they are involved in the synthesis of commercially important compounds [47–50]. Understanding their heterologous expression properties and enzymatic characteristics can enhance terpenoid productivity and commercial availability via successful metabolic engineering approaches.

## Methods

### Plant material and microbial strains

NEB10β *Escherichia coli* cells (New England Biolabs) were used for all sub-cloning steps. Respective constructs were transformed into chemically competent NEB10β *E. coli* cells and transformants were selected on 50 g/L Kanamycin LB plates. The positive transformants were screened using PCR and grown overnight in 50 g/L kanamycin LB medium. Plasmid DNA was isolated using the QIAprep Spin™ miniprep Kit (Qiagen) according to provider’s manual. *A. tumefaciens* PGV 3850 cells were transformed via electroporation as described in [51]. Positive transformants were selected on YEP plates with 50 g/L kanamycin and 25 g/L rifampicin, incubated at 28 °C for two days. *N. benthamiana* plants were grown in greenhouse conditions (Day temperature 25 °C; Night temperature 20 °C; 16 h light/8 h dark cycle; light intensity of 200 – 600 μmol photons .m^−2^.s^−1^) in peat and were infiltrated with transformed *A. tumefaciens* as described in [15]. The infiltrated plants grew in the greenhouse for four days, followed by harvesting of leaf material for further analysis.

### Sequences and constructs

The *P19* silencing repressor gene [GenBank: NP_612584.1] [29] was cloned into pCAMBIA1300 vector (pLIFE-33) and the *diTPS-ISO* gene from *Picea abies* [GenBank: AY473620.2] was cloned with a C-terminal FLAG tag into pEAQ-HT vector [52]. The *DXS* and *GGPPs* [GenBank: KP889114 and KP889115 respectively] coding regions from *P. barbatus* were also inserted into the pCAMBIA vector. *CYP720B4* coding sequence from *Picea sitchensis* [GenBank: HM245403.1], Barnes-CYP and ΔTm-CYP coding sequences were inserted into an AgeI-XhoI linearized pEAQ-HT vector via T4 ligation (T4 ligase from New England Biolabs). The transit peptide (Tp) sequence coding for the N-terminal 52 amino acid of the Arabidopsis ferredoxin protein FedA [GenBank: AY128936] was amplified from the template *Fd-CYP79A1* construct in pEAQ-HT mentioned in [15] and was introduced upstream of the *CYP720B4* coding sequences using overlap-extension PCR [53]. The primers used in this work are listed in Table 1.

**Table 1:** PCR primers

Name	Sequence (5'-3')	Comment
P1	TCGCGACCGGTAATGGCGCCCATGGC	CYP720B4 forward primer
P2	ATCTCGAGTTATTCATTCTCTACTCTACCATGAAG	CYP720B4 reverse primer
P3	GCGACCGGTAATGGCTCTGTTATTAGC	Barnes-CYP720B4 forward primer
P4	TCGCGACCGGTAATGAGTAATAAGGAGGT	ΔTm-CYP720B4 forward primer
P5	CGACCGGTAATGGCTTCCACTGCTC	Tp forward primer
P6	AGCGGCTGTGACACG	Tp reverse primer
P7	TCGTCTTTGTAGTCTTCATTCTCTACTCTAC	CYP720B4 reverse primer, containing FLAG tag

### GC-MS analysis

For GC-MS analysis, four leaf discs (Ø = 3 cm) from *N. benthamiana* plants infiltrated with the respective constructs were taken. The cut leaves were incubated in 1.5 ml glass vials containing pure hexane (GC-MS grade) spiked with the internal standard (0.1 mg/ L eicosene) for 1 h at room temperature at the Roto-Shake Genie® (Scientific Industries, Inc.) revolving at 25–30 cycles/min.

Extracts were analyzed on a Shimadzu GCMS-QP2010 Ultra using an HP-5MS column (20 m x 0.180 mm i.d., 0.18 μm film thickness, Agilent technologies). Injection volume and temperature were set at 1 μL and 250 °C in splitless mode (GC program: 60 °C for 1 min, ramp at rate 20 °C min-1 to 160 °C, ramp at rate 5 °C min-1 to 240 °C, ramp at rate 20 °C min-1 to 320 °C and hold for 2 min). H_2_ were used as carrier gas with a linear velocity at 66.5 cm s-1 and a purge flow of 4 mL min-1 for 1 min. Ion source temperature of the mass spectrometer was set to 280 °C and spectra’s was recorded from m/z 50 to m/z 350 with a solvent cutoff at 4 min. Compound identification was done with comparison to authentic standard of isopimaradiene as described in [26].

Isopimaradiene was quantified on a SCION 436 GC-FID (Bruker). 2 μL sample was injected in splitless mode at 280 C° (GC program same to the one mentioned above). After the transfer of the sample from the injection port to the HP5 column (1 min), the injection port was set to split 1:50. H_2_ was used as carrier gas with a constant flow of 1 mL min-1. The FID was set at 300 °C, with a N_2_ flow of 25 mL min-1, H_2_ at 30 mL min-1 and air 300 mL min-1. Data sampling rate was taken at a frequency of 10 Hertz. Isopimaradiene was identified based on the retention time compared to the authentic standard. Quantification of isopimaradiene was done by integration of the isopimaradiene peak area and calculated from the concentration of the internal standard. The relative response factor between isopimaradiene and the internal standard was set to 1.

### LC-MS analysis

Four leaf discs per plant were cut, flash-frozen in liquid nitrogen, and homogenized in 80 v/v % methanol aqueous solution. Samples were diluted with water to 50 v/v % methanol final concentration and briefly spun down in mini-centrifuge. 40 μL of the supernatant was used for LC-MS analysis. The LC-MS analysis was performed as previously described [54] with the gradient program set as: 0 to 1 min, isocratic 50 % B; 1 to 10 min, linear gradient 50 to 95 % B; 10 to 11.4 min, isocratic 98 % B; 11.4 to 17 min, isocratic 50 % B.

### Pigment analysis

The pigments composition of leaves was analyzed immediately after extraction in acetone/H_2_O/25 % NH_4_OH (80/20/1 vol/vol/vol) as described in [55, 56] with minor modifications. The Dionex HPLC system with a Supelco LICHROSPHER RP18-5 analytical (250 mm x 4.6 mm inner diameter; 5 μm particle size) was used. The mobile phase consisted of two solvents, A: acetonitrile/methanol/0.1 M Tris–HCl pH 8.0 (84/2/14 vol/vol/vol) and B: methanol/ethyl acetate (68/32 vol/vol). The pigments were eluted with a linear gradient from 100 % solvent A to 100 % solvent B over 12 min, followed by an isocratic elution with 100 % solvent B for 6 min, and a linear gradient of 100 % solvent B to 100 % solvent A in 1 min. The column was regenerated with 100 % solvent A for 11 min before injection of the next sample. Injection volume was 40 μl, the flow rate was 1 ml/min, and peaks were detected and integrated at 445 nm for carotenoid and chlorophyll content. Pigments were identified by comparing retention times and absorption spectra with standard pigments (DHI, Hørsholm, Denmark). Quantification was performed by integration of the elution peaks at 445 nm using the program Chromeleon version 6.7 (Dionex, Sunnyvale, CA).

### Immunoblotting

10 mg of flash-frozen leaf material was grinded using a ball mill (Retsch, US) (Pulse 30 and 1.5 min, 3 times). To the crushed leaf material 100 μL of SDS-PAGE sample buffer (Bio-Rad) was added and vortexed for 2 mins. The vortexed mixture was centrifuged at 10000 g for 10 mins. The supernatant was recovered and used for gel electrophoresis on 12 % SDS Criterion™ TGX Stain-Free™ Precast Gel (Bio-Rad). Immunoblotting was performed as described in [15]. Proteins were transferred to PVDF membrane using the Trans-Blot® Turbo™ Transfer Starter System (Bio-Rad) according to manufacturer’s protocol. The primary antibodies used were monoclonal anti-FLAG M2 antibody (Sigma-Aldrich Cat# F3165, RRID: AB_259529) of 1:1000 dilution in 5 % skimmed milk PBS-T. As the secondary antibody, polyclonal rabbit anti-mouse/HRP (DAKO Cat# P 0447) of 1:2000 dilutions in 5 % skimmed milk PBS-T was used. Gels and membranes were visualized in the ChemiDoc™ MP System (Bio-Rad). Total chlorophyll and chlorophyll a/b ratios were determined in 80 % acetone as described in [57].

## Abbreviations

DXS: 1-deoxyxylulose-5-phosphate synthase
ER: Endoplasmic reticulum
GC-MS: Gas chromatography/mass spectrometry
GGPP: Geranylgeranyl diphosphate
GGPPs: GGPP synthase
HPLC: High performance liquid chromatography
LC-MS: liquid chromatography/mass spectrometry
